# Hybrid optimized remaining useful life prediction framework for lithium-ion batteries with limited data samples

**DOI:** 10.1038/s41598-025-26743-1

**Published:** 2025-11-07

**Authors:** Md Ibrahim, Shaheer Ansari, Afida Ayob, M. S. Hossain Lipu, Maher G. M. Abdolrasol, Abdul Waheed Khawaja, Muhammad Amir Khalil, Daniel Ioan Stroe

**Affiliations:** 1 Department of Electrical Engineering, Integral University, Lucknow, 226026 India; 2 Department of Engineering, School of Engineering and Technology, Sunway University, Petaling Jaya, 47500 Selangor Malaysia; 3 Centre for Integrated Systems Engineering and Advanced Technologies (INTEGRA), Faculty of Engineering and Built Environment, Universiti Kebangsaan Malaysia (UKM), Bangi, 43600 Malaysia; 4 Department of Electrical Electronic and Systems Engineering, Universiti Kebangsaan Malaysia, Bangi, 43600 Selangor Malaysia; 5 School of Electrical Computer and Telecommunications Engineering, University of Wollongong, Wollongong, NSW 2522 Australia; 6 Institute of Sustainable Energy, Universiti Tenaga Nasional, Kajang, 43000 Selangor Malaysia; 7 Department of Electrical Engineering, Faculty of Engineering & Technology, Bahauddin Zakariya University, Multan, 60800 Pakistan; 8 Faculty of Engineering, Multimedia University, Cyberjaya, 63100 Selangor Malaysia; 9 Department of Energy, Aalborg University, Aalborg, 9220 Denmark; 10 Marine Engineering Department, Military Technological College, Muscat, Oman

**Keywords:** Lithium-ion battery, Remaining useful life, Optimization, Data-driven, Engineering, Electrical and electronic engineering

## Abstract

This study introduces a Jellyfish optimization technique integrated with a Multi-Layer Perceptron, specifically a Feedforward Neural Network (FNN) model, for remaining useful life (RUL) prediction of lithium-ion batteries (LIBs). A multiple battery with multi-input (MBMI) profile is utilized to create 91-dimensional data features for model training. A systematic sampling approach is employed to extract relevant data features. Results show that the proposed JFO-based FNN model outperforms the traditional FNN model’s accuracy. The Mean Square Error (MSE) is used as the objective function to determine optimal model hyperparameters. The research utilizes the NASA LIB database, which includes four datasets. For LIB cell B5, the proposed model achieved an MSE of 3.9494*10^− 4^. The model’s accuracy and efficiency are further validated using particle swarm optimization. However, the LIBs B6 and B18 showed higher error results due to capacity regeneration issues. The MIT-Stanford LIB datasets demonstrated high applicability when validating the JFO-FNN model’s outcomes. The novelty of this work lies in using a JFO-optimized FNN model trained on systematically sampled, multi-battery LIB datasets to improve predictive accuracy, generalization, and robustness. Overall, the developed RUL prediction framework appears to be fast, effective, and yields promising results.

## Introduction

Presently, a wide range of industries, including communication, energy management systems, electric cars, and aircraft, use lithium-ion batteries (LIBs)^1–3^. Low voltage drop, high energy density, high electromotive force, ease of handling, and a broad operational temperature range are just a few of the benefits that LIBs offer in a variety of applications^4,5^. Repeated charge and discharge cycles already lead to the aging of LIBs and reduced health. It leads to serious consequences, including degradation through failure of the system, breakdown, and economic loss^6^. It is emphasized to be necessary for the control and maintenance of the LIBs health to deliver efficient performance throughout its life^7^. The controlled operations of the LIBs avoid problems of degradation, however, it is necessary to predict the remaining useful life of the LIB precisely to reduce the risk of unexpected LIB failure, optimize resources, and thereby get effective and reliable LIB performance^8^.

The use of LIBs in different applications decreases their capacity. The LIB is replaced for safety reasons when the capacity nears a threshold limit set at around 70% to 80% of the rated original capacity^9^. Therefore, it is imperative to closely predict the remaining useful life of the LIB to ensure the safe and reliable operation of the energy storage system (ESS). The accurate evaluation mitigates safety concerns and ensures that LIB functions reliably and efficiently. The RUL prediction is the number of charging and discharging cycles that the LIB can undergo before it arrives at the threshold level and is calculated using its present cycle.

There are two main approaches to forecasting the RUL i.e. model-based and data-driven methods. Mathematical modelling and derivations that require empirical and experimental validation are considered with model-based methods^9^. These techniques use different sets of equations to define the LIBs’ behavior and calculate the RUL. In^10^, the authors proposed the RUL prediction algorithm using the unscented particle filter (PF). The issue with sample degeneracy associated with UPF was minimized with the implementation of the Markov Chain Monte Carlo method. Furthermore, three models were reported by Walker et al.^11^ including nonlinear least squares, particle filter, and unscented Kalman filter. The models correctly predict RUL, whereas model-based methods need some degree of information that can precisely describe the internal workings to enhance RUL prediction accuracy.

The data-driven approaches for estimating the RUL derive parameters, such as capacity, impedance, voltage and current. Data-driven models outperform model-based models in accuracy, speed, and simplicity^12^. For instance, a Support Vector Machine (SVM) model to assess the LIB’s health and prognosis was proposed^13^. This model combined with the PF technique to show the LIB’s degradation profile. Yet, the SVM-PF model’s training used only capacity data, resulting in low-dimensional training data and less precise RUL results. Liu et al.^14^ later introduced the Relevance Vector Machine (RVM) algorithm, which incorporates an online training method to improve RUL prediction accuracy using the NASA database. Using an ANN model, Ansari et al.^15^ employed the back propagation neural network (BPNN) model to predict the RUL. The model training was performed with data collected through a mathematical sampling technique. Nevertheless, the execution time was high and further requires human experience in choosing the appropriate model parameters. In^16^, this issue was addressed by appropriately selecting the model parameters using a bat-based PF model. The method demonstrated effective outcomes and versatility to varying dynamic trends. Table 1 delivers a comparative evaluation of literature research. Additionally, Ansari et al.^17^ introduced a new RUL prediction algorithm consisting of bio-inspired jellyfish optimization (JFO) and FNN model. The JFO selects the FNN model parameter to achieve satisfactory results, nonetheless, LIB datasets with varying characteristics were not considered to validate the developed model.

The aforementioned studies fall short in accuracy due to the absence of data variety from multiple operational profiles, resulting in the inability to accurately predict the RUL. Moreover, the model parameters were inadequately chosen for training. Henceforth, this study presents the RUL prediction of the LIB using an optimized model with systematic sampling to precisely attain the data and accomplish accurate model training and further validate the proposed model with the LIB database with dynamic characteristics. Furthermore, in contrast to other studies that focus on single battery single input (SBSI) profiles and single inputs^18,19^, this approach considers MBMI profile factors. These include the acquisition of data parameters from multiple LIBs to attain higher data dimensionality. The mathematical sampling process is applied to select 30 samples of the LIB parameters like voltage, current, and temperature (VIT). Additionally, the capacity data is added to develop the proposed MBMI data framework. Lastly, the FNN model parameters are accurately optimized with the JFO. The key novelty of the paper is as follows:

- Implementation of the JFO algorithm for selecting optimal model hyperparameters, including hidden neurons and learning rate, to enhance predictive accuracy and computational efficiency.
- Utilization of multi-battery parameters to construct a comprehensive and suitable data framework for efficient model training and generalization across different LIB datasets.
- 30 data samples are carefully selected using the systematic sampling (SS) technique to ensure optimal representation and accuracy in model training.
- The optimized FNN model is trained with various LIB datasets to enhance its predictive performance and robustness, across different LIB characteristics.

The paper’s structure is as follows: Section II discusses LIB data acquisition. Section III delivers the methodology and proposed framework for the RUL prediction. Section IV details the experimental results, with the conclusion in Section VI.

**Table 1:** Comparative analysis of literature works for rul prediction of the lib.

Ref.	Model	Parameters used	Strength	Weakness
^10^	UPF	Capacity	Low computational complexity with more accurate RUL prediction outcomes	Noise inclusion in the data was not considered which could increase the characteristics of the data
^11^	UKF-PF	Capacity	The RUL prediction outcome demonstrated high applicability of the proposed model	The parameters from the operating profile were not used to develop the data framework
^13^	SVR	Capacity and impedance	The generalization ability of the model was high	The selection of model parameters required trial and error which needed human expertise and time
^14^	RVM	Mean voltage falloff	The RUL prediction outcomes were accurate. The reconstitution of the capacity degradation curve is accurate	The Mean voltage falloff can be obtained only with stable operating conditions and not with a transient operation state such as EV
^15^	BPNN	Capacity, voltage, current, temperature	Different LIB parameters were included to train the BPNN model	The selection of the BPNN model parameters was time-consuming
^16^	FNN-PF	Capacity	Model parameter selection was conducted with an optimized technique	The model training was not comprehensive based on different training ratios
^17^	FNN-JFO	Capacity, voltage, current, temperature	Selection of features using a mathematical sampling method	-The model was not validated with other LIB datasets

### Lithium-ion battery data acquisition

The experimental LIB dataset is sourced from the NASA Prognostics Centre of Excellence Data Repository, which offers four datasets from Li-ion 18,650-sized rechargeable LIBs^23^. The LIB parameters are analyzed, and their data structure is established using the constant current and constant voltage (CCCV) method. Three distinct operating profiles (charge, discharge, and electrochemical impedance spectroscopy) were applied to LIBs at varying temperatures. Various current load levels were used during discharges before the LIBs voltage dropped to predetermined thresholds.

**Fig. 1:**
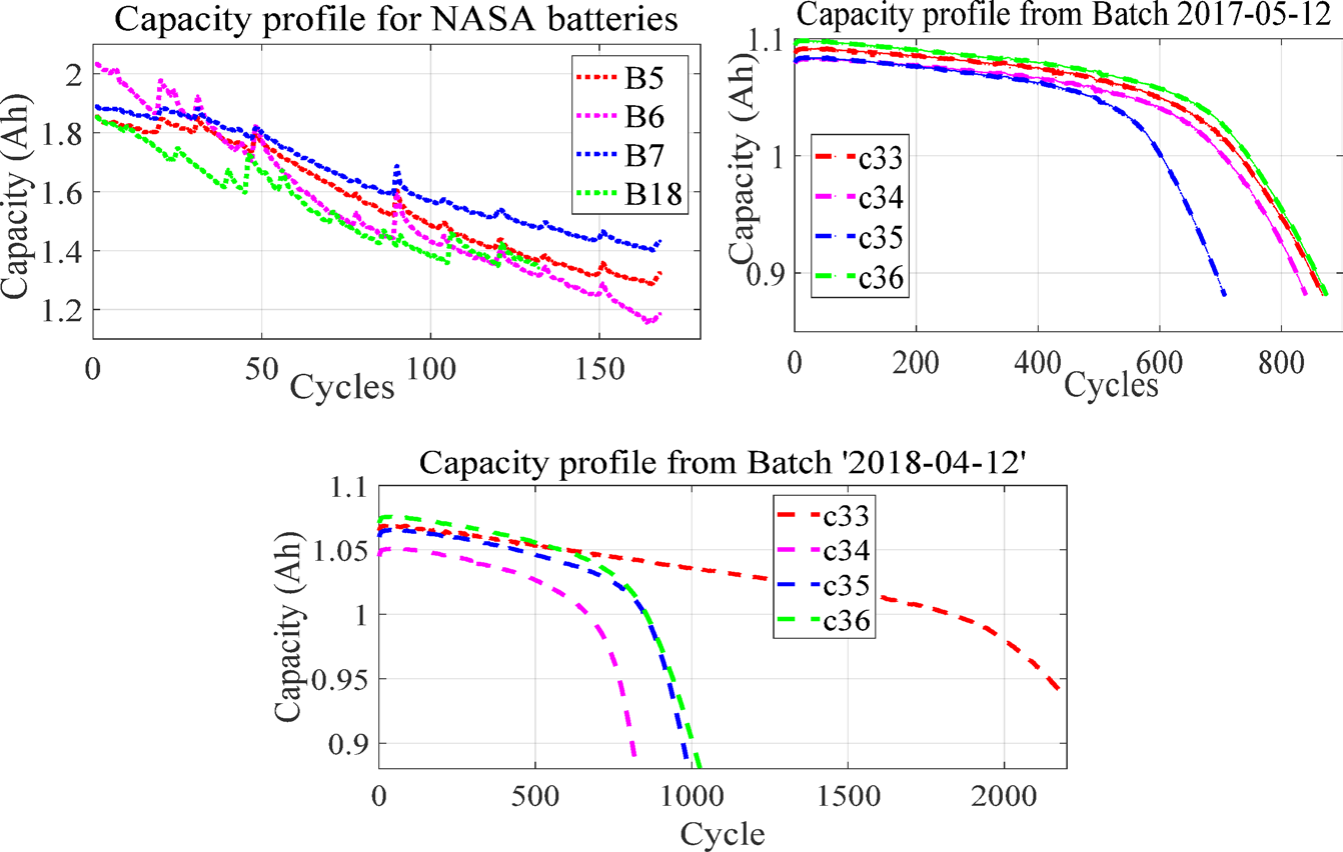
Capacity degradation curve of NASA and MIT-Stanford LIB dataset (Two batches i.e. ‘2017-05-12’ and ‘2018-02-20’).

Additionally, the JFO-FNN model is validated with the MIT-Stanford LIB dataset from two batches i.e. ‘2017-05-12’ and ‘2018-04-20’^20^. Each LIB cell has a nominal capacity and nominal voltage of 1.1 Ah and 3.3 V. For batch ‘2017-05-12’, all cells were charged in cycles using either a one-step or two-step procedure. The range of the charging time is around 8 to 13.3 min (0–80% SOC). Except for 3.6 C (80%), two cells are typically tested per policy. A rest period of 1 min and 1 s was implemented after achieving 80% state of charge during charging and after the discharging operation. The LIB cells were cycled to 0.88 Ah, or 80% of their nominal capacity. Whereas, for the batch ‘2018-04-12’, every LIB cell was cycled using a two-step charging operation. A 10-minute charging duration is set for each cell. A 5-second rest time was applied after four operations i.e. before and after discharging, after internal resistance test and after attaining 80% SOC while charging. A random selection of the cell based on the previously accomplished work is undertaken and the dataset from four cells namely c33, c34, c35 and c36 is acquired from two batches^21,22^. It is noted that the MIT-Stanford dataset shows dynamic features whereas, the NASA dataset is constructed under non-dynamic operating conditions. On the other hand, the current value fluctuates rapidly over time during the discharging operation, making it difficult to accurately obtain the LIB data parameters. Additionally, because no preassigned methods are used throughout the discharging operation, the LIB parameters exhibit a considerable degree of unpredictability. The LIB parameters from the discharge profile are not taken into account because of this concern^23^. The capacity degradation profile of the NASA and MIT-Stanford LIB dataset is shown in Fig. 1. Additionally, the specifications of the LIB cells with their source, charging/discharge protocol and cycle rate is presented in Table 2.

**Table 2:** Description of the lib characteristics for Nasa and mit-stanford database.

LIB cell	Source	Charging protocol	Discharge protocol	Cycle rate
B5	NASA	Constant Current (1.5 A) + Constant Voltage (4.2 V)	Constant Current (2 A) to 2.7 V	1 C
B6				
B7				
B18				
C33	MIT-Stanford “2017-05-12”	4 C constant current to 4.2 V	4 C to 2.8 V	4 C
C34				
C35				
C36				
C33	MIT-Stanford “2018-04-12”	1 C constant current to 4.2 V	1 C to 2.8 V	1 C
C34				
C35				
C36				

## Rul prediction methodology and framework

The presented methodology and framework were constructed using the FNN model, a bio-inspired algorithm named the JFO technique and the SS method. First, the FNN model is used in a wide range of applications, including face recognition, simulation modelling, and time series prediction, to mention a few. The popularity of these applications has generated interest in creating effective training techniques that have quick convergence and strong generalization capabilities^24^. Recently, the JFO approach has been designed and has been used in numerous research projects^25^. The JFO approach demonstrates an excellent ability to solve optimization problems and is simple to use. Currently, the JFO technique has not been used to a greater extent for state estimation of the LIB but except few works published^21,26^. Consequently, the JFO technique is taken into consideration when developing the suggested RUL prediction framework to appropriately select the FNN model parameters. Finally, systematic sampling is employed to identify the precise data characteristics of key parameters to establish a suitable data structure.

### Multilayer perceptron-based feedforward neural network

**Fig. 2:**
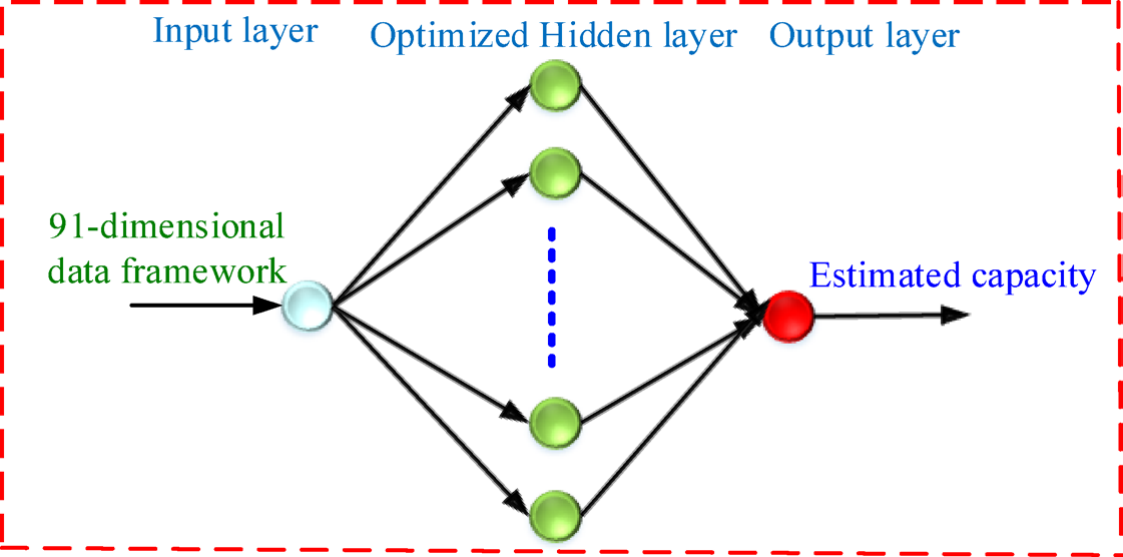
Proposed MLP based FNN model for the RUL prediction.

The Multilayer perceptron (MLP) based FNN model is commonly applied in machine learning systems because of its easy implementation and nonlinear response^27^. The paper proposes a three-layer FNN model consisting of the input layer, hidden layer and output layer as depicted in Fig. 2. The input layer takes the constructed 91-dimensional data, the hidden layer is optimized and the output layer delivers the estimated capacity to attain the required RUL prediction results. Moreover, the Levenberg-Marquardt Algorithm for training the FNN model is considered^15^. The FNN model has an input layer, which considers the input from the selected LIB parameters. The JFO technique determines the hidden neurons and learning rate in the FNN model. However, selecting the conventional FNN model parameters is undertaken with a trial-and-error technique. Parameters such as learning rate, hidden neurons, number of epochs and iterations are selected with a trial-and-error technique. Table 3 depicts the selection of the conventional FNN model hyperparameters. The selection of the model hyperparameters is considered with the training dataset as B6, B7 and B18 while the testing dataset is taken as B5. It was examined that minimum RMSE was achieved with a hidden neuron of 5, learning rate of 0.01, number of epochs as 500 and number of iterations as 50 respectively. The learning rate i.e. 0.01 indicates the slow learning ability of the FNN model but with better accuracy and prediction outcomes.

**Table 3:** Selection of the Fnn model parameters with the trial and error method.

Hidden neurons	Learning rate	Iterations	Epochs	RMSE
10	0.001	100	1000	0.3246
20	0.0001	200	2000	0.7787
30	0.1	50	500	0.6624
5	0.001	100	1000	1.8291
10	0.001	100	2000	0.2165
15	0.01	50	500	0.3476
20	0.001	100	1000	0.4120
**5**	**0.01**	**50**	**500**	**0.1781**

The anticipated capacity is the only neuron that the FNN model’s output layer contains. The output and hidden layers are regarded as processing layers in the FNN model. These layers can be activated using a hyperbolic tangent sigmoid function. The activation function is expressed as follows:

1 $$\:f\left(u\right)=\frac{2}{1+{e}^{-2u}}-1$$

The hidden layer in the FNN model is expressed as follows:

2 $$\:{H}_{i}=f(\sum\:_{i=1}^{n}{V}_{i}{W}_{i}^{H}+{b}_{j}^{H}$$

refers to the number of hidden neurons. The expression of the output based on the output layer of the FNN model can be expressed as:

3 $$\:No=f(\sum\:_{j=1}^{n}Hf{W}_{j,k}^{o}+{b}_{k}^{o}$$

where, *n* denotes the hidden neurons, $\mathop W\nolimits_{{j,k}}^{o}$ is the weight between the $jth$ hidden neuron and output neuron, $\mathop b\nolimits_{k}^{o}$ express the threshold value for $kth$ output neuron.

### Bio-inspired jellyfish optimization (JFO) technique

The bio-inspired algorithm i.e. JFO technique was invented in the year 2020 and depicts the jellyfish movement to search best outcomes. The JFO techniques find the solutions by considering many factors such as ocean currents, jellyfish movement, time control mechanisms, and jellyfish bloom. The JFO was selected due to its dynamic balance between exploration and exploitation phases, inspired by real biological behavior, which is particularly effective for non-convex and high-dimensional search spaces such as hyperparameter tuning in neural networks. Unlike Genetic Algorithms, JFO requires fewer control parameters and avoids premature convergence. Compared to Bayesian Optimization, which is computationally expensive due to surrogate modeling, JFO offers a light but competitive alternative. Additionally, Grid Search, while thorough, is not practical in high-dimensional or continuous spaces. Consequently, JFO strikes a good balance between efficiency and effectiveness in optimizing the learning rate and hidden neurons of the suggested FNN model. The JFO algorithm follows three search operational principles, namely:

1. *Ocean current*

The abundance of food in the ocean currents attracts jellyfish. The average of all vectors from jellyfish to jellyfish at the ideal point is used to illustrate the ocean trend.

4 $$\:\stackrel{-}{trend}=\frac{1}{npop}\sum\:{trend}_{i}=\frac{1}{npop}\sum\:\left({X}^{*}-{e}_{c}{X}_{i}\right)={X}^{*}-{e}_{c}\frac{\sum\:{X}_{i}}{npop}{X}^{*}{-e}_{c}\mu\:={X}^{*}-d$$

where, $df=\mathop e\nolimits_{c} \mu$ and is the deviation between the mean location of all jellyfish and their current ideal position, $\mathop n\nolimits_{{pop}}$ denotes jellyfish population,$\mathop X\nolimits^{*}$ indicates the ideal location within the swarm, $\mu$is the mean position of the jellyfish and $\mathop e\nolimits_{c}$is a factor controlling pull and.

2) *Jellyfish swarm*

The collective jellyfish movement starts as passive (Type A) and, over time, accelerates into active movement (Type B). In their passive state, jellyfish are assumed to move in a circling pattern around their current positions, and the updated position is given by:

5 $$\:\overrightarrow{{X}_{i}}\left(t+1\right)=\overrightarrow{{X}_{i}}\left(t\right)+\overrightarrow{rand}*\delta\:*\left({H}_{b}-{L}_{b}\right)$$

where $\mathop H\nolimits_{b}$and $\mathop L\nolimits_{b}$express the maximum and minimum limits of the boundary conditions in the search space. The coefficient of motion is $\delta$> 0.

3) *Time control mechanism*

The jellyfish’s movement and timing are modeled using a time control mechanism that governs its motion in ocean currents. This mechanism consists of a time-dependent function, c(t), and a constant. The function c(t) is random and takes on values between 0 and 1 over time, and it is expressed as:

6 $$\:c\left(t\right)=\left(1-\frac{t}{{T}_{max}}\right)*\left(2*rand\left(\text{0,1}\right)-1\right)$$

The described structure is well in place, JFO techniques used for selecting the most appropriate FNN model hyperparameters, obeying optimization constraints. It utilizes a correlated search space for identifying the optimal FNN parameters, with this space limited by the minimum and maximum values set for the parameters to be optimized. Establishing accurate boundaries is crucial for selecting the proper hyperparameters; otherwise, deviations may occur, resulting in suboptimal predictions. Nonetheless, the effectiveness of the JFO technique is evaluated with Mean Square Error (MSE) as the objective function and denoted as:

7 $$\:MSE=\left(\frac{1}{n}\sum\:_{n=1}^{n}{\left|ck-{ck}^{\varDelta\:}\right|}^{2}\right)$$

where $ck$ represents the real capacity data samples, $\mathop {ck}\nolimits^{\Delta }$ denotes the predicted capacity data samples. Table 4 shows the model parameters applied to the proposed JFO-FNN model are presented. The selection of the parameters such as number of epochs, iterations and population size is determined using the trial-and-error method. It is examined that the model hyperparameters of FNN should be carefully selected. For instance, a model with a high learning rate could miss the optimal values, whereas a low learning rate could lead to slow convergence. Overfitting might result from using too many layers, while modeling difficult data wouldn’t be possible with too few.

**Table 4:** Model hyperparameters for the proposed jfo-fnn model.

JFO-FNN model hyperparameters	Assigned values
Population size	5
Number of iterations	100
Number of epochs	1000
Lower bound for hidden neurons	1
Upper bound for hidden neurons	40
Lower bound for learning rate	0
Upper bound for learning rate	1

The lowest and maximum values of the model’s hyperparameters that need to be carefully optimized define the boundaries of the search space used for choosing the optimal FNN parameters. For instance, a boundary condition [1 40] was assigned for hidden neurons whereas, [0 1] was assigned to achieve an optimized learning rate. It is crucial to choose the hyperparameters with an appropriate boundary; otherwise, the results might diverge significantly and produce unsatisfactory prediction results. The selection of the different JFO parameters is shown in Table 5.

**Table 5:** Model hyperparameter selection for jfo-fnn model.

Population size	Iterations	Epochs	RMSE
3	50	500	1.0290
3	100	1000	0.0719
3	200	2000	0.0323
5	50	500	0.4683
**5**	**100**	**1000**	**0.0292**
5	200	2000	0.4403

**Fig. 3:**
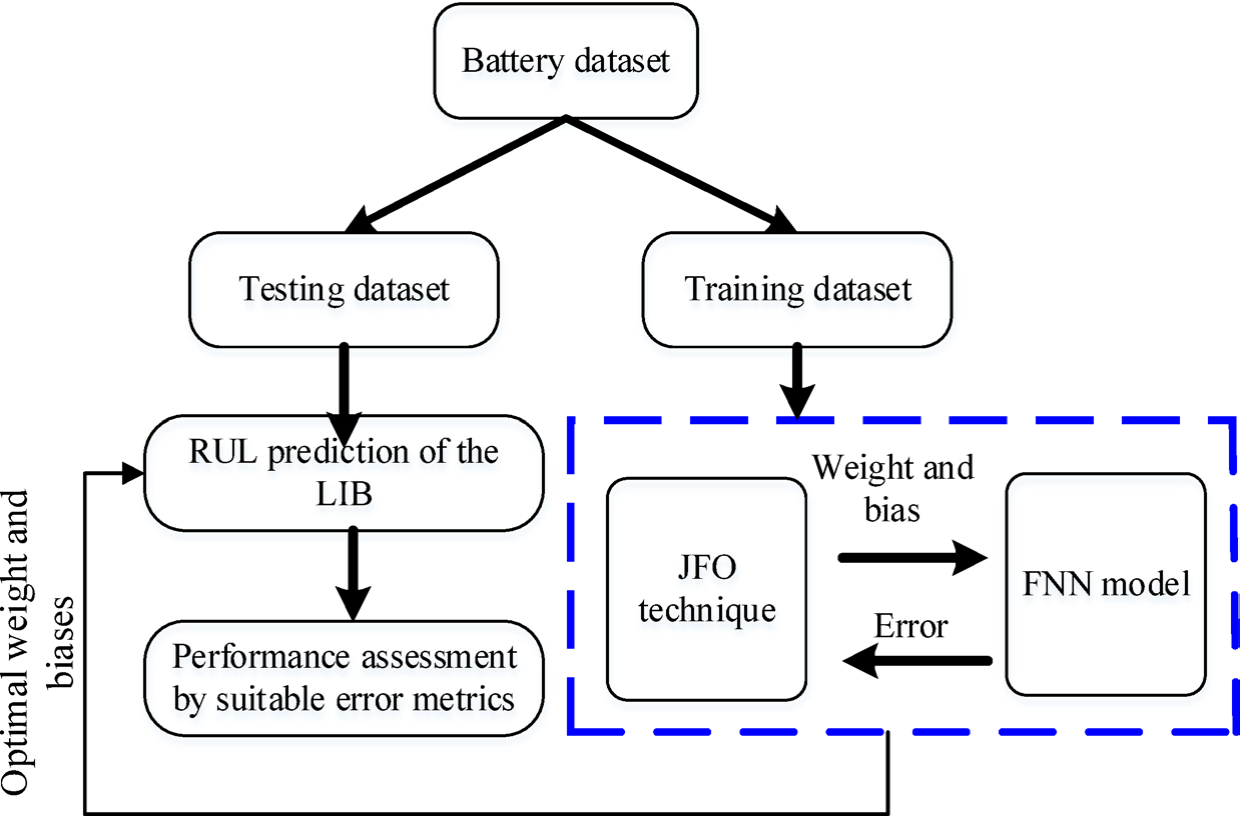
Training process of the JFO-FNN to achieve optimized weight and bias.

The evaluation to choose the JFO parameters is performed on the preliminary studies, where B5 is selected as a testing dataset and batteries B6, B7, and B18 were training datasets. Henceforth, the precise selection of the optimization parameters is crucial as it leads to better model training with higher generalization and lower computational complexity. Figure 3 illustrates the fundamental structure of the FNN model training with JFO. The movement of each jellyfish inside the swarm is updated using the time control mechanism. The updating continues until the best objective function is attained. The optimized hyperparameters of the FNN model are generated by JFO. The objective function determined by the FNN model is returned as the fitness value for the JFO. The best jellyfish within the operation is the ideal result, and the FNN model makes use of it.

### Data extraction with a mathematical sampling method

The mathematical sampling method applied in this work is referred to as systematic sampling (SS). The SS method is considered to extract the features/samples by considering an equal time interval from a large dataset^28^. The equal time interval is the sampling interval and is obtained by dividing the total data features/samples by a suitable sampling size. The sample size is selected by considering the model’s operation, constraints and outcomes. It is important to understand the suitable selection of the data sample size. A large number of data samples could result in low sampling error and computational complexity whereas, a small data size may lead to non-sampling error. The selected data samples from the parameter profile show the approximate characteristics of the parameter. However, some relevant features having a strong correlation with the parameter characteristics may be left unnoticed. Therefore, the sample size in the SS technique should be accurately calculated so that important parametric features are attained. The SS method reduces the likelihood of utilizing filter methods to eliminate the unwanted/noisy data and avoids the need for clustered data^19^.

In the proposed work, the SS method is utilized to attain 30 data samples from each parameter i.e. VIT and one sample from capacity data. The initial evaluation with different data features suggested the accurate data feature size as 30. The combined 30 samples from VIT and a single capacity sample formed a 91-dimensional data structure. The proposed 91-dimensional data framework created with the SS technique is as follows:

8 $$\:I=\left[\begin{array}{c}\left\{\frac{{V}^{a}}{30}*1,\frac{{V}^{a}}{30}*2\dots\:\frac{{V}^{a}}{30}*30\right\}\left\{\frac{{t}^{a}}{30}*1,\frac{{t}^{a}}{30}*2\dots\:\frac{{t}^{a}}{30}*30\right\}\left\{\frac{{i}^{a}}{30}*1,\frac{{i}^{a}}{30}*2\dots\:\frac{{i}^{a}}{30}*30\right\}\left\{{C}_{a}\right\}\\\:\left\{\frac{{V}^{b}}{30}*1,\frac{{V}^{b}}{30}*2\dots\:\frac{{V}^{b}}{30}*30\right\}\left\{\frac{{t}^{b}}{30}*1,\frac{{t}^{b}}{30}*2\dots\:\frac{{t}^{b}}{30}*30\right\}\left\{\frac{{i}^{b}}{30}*1,\frac{{i}^{b}}{30}*2\dots\:\frac{{i}^{b}}{30}*30\right\}\left\{{C}_{b}\right\}\\\:.\\\:.\\\:.\\\:\left\{\frac{{V}^{n}}{30}*1,\frac{{V}^{n}}{30}*2\dots\:\frac{{V}^{n}}{30}*30\right\}\left\{\frac{{t}^{n}}{30}*1,\frac{{t}^{n}}{30}*2\dots\:\frac{{t}^{n}}{30}*30\right\}\left\{\frac{{i}^{n}}{30}*1,\frac{{i}^{n}}{30}*2\dots\:\frac{{i}^{n}}{30}*30\right\}\left\{{C}_{n}\right\}\end{array}\right]$$

where, *a*, *b* and *n* represents the cycle 1, cycle 2 and last cycle. For $\:\left\{\frac{{V}^{a}}{30}*1,\frac{{V}^{a}}{30}*2\dots\:\frac{{V}^{a}}{30}*30\right\}$, $\:\frac{{V}^{a}}{30}*1\:$represents the first voltage feature, $\:\frac{{V}^{a}}{30}*2$ is the second data feature and $\:\frac{{V}^{a}}{30}*30$ is last voltage sample from first charging cycle. Similarly, the data samples are extracted from the temperature and current profiles. Additionally, $\:{C}_{a}$, $\:{C}_{b}\:$and $\:{C}_{n}\:$are the first, second and last extracted capacity samples. The proposed developed data framework depicts the input data samples as *I* and target data samples i.e. *C* for proposed JFO-FFN model can be shown as:

$$\:I=\left[\begin{array}{c}\left\{\frac{{V}^{1}}{30}*1,\frac{{V}^{1}}{30}*2\dots\:\frac{{V}^{1}}{30}*30\right\}\left\{\frac{{t}^{1}}{30}*1,\frac{{t}^{1}}{30}*2\dots\:\frac{{t}^{1}}{30}*30\right\}\left\{\frac{{i}^{1}}{30}*1,\frac{{i}^{1}}{30}*2\dots\:\frac{{i}^{1}}{30}*30\right\}\left\{{C}_{1}\right\}\\\:\left\{\frac{{V}^{2}}{30}*1,\frac{{V}^{2}}{30}*2\dots\:\frac{{V}^{2}}{30}*30\right\}\left\{\frac{{t}^{2}}{30}*1,\frac{{t}^{2}}{30}*2\dots\:\frac{{t}^{2}}{30}*30\right\}\left\{\frac{{i}^{2}}{30}*1,\frac{{i}^{2}}{30}*2\dots\:\frac{{i}^{2}}{30}*30\right\}\left\{{C}_{2}\right\}\\\:.\\\:.\\\:.\\\:\left\{\frac{{V}^{n}}{30}*1,\frac{{V}^{n}}{30}*2\dots\:\frac{{V}^{n}}{30}*30\right\}\left\{\frac{{t}^{n}}{30}*1,\frac{{t}^{n}}{30}*2\dots\:\frac{{t}^{n}}{30}*30\right\}\left\{\frac{{i}^{n}}{30}*1,\frac{{i}^{n}}{30}*2\dots\:\frac{{i}^{n}}{30}*30\right\}\left\{{C}_{n}\right\}\end{array}\right],\:C=\left[\begin{array}{c}C1\\\:C2\\\:.\\\:.\\\:.\\\:Cn\end{array}\right]$$

where $\:\left\{\frac{{V}^{1}}{30}*1,\frac{{V}^{1}}{30}*2\dots\:\frac{{V}^{1}}{30}*30\right\}\:$denotes the voltage samples from cycle 1, cycle 2 and the last cycle. A similar method is applied to the temperature and current parameters. $\:C1,\:C2\dots\:Cn$ signifies estimated capacity from cycle one to cycle n i.e. last cycle.

**Fig. 4:**
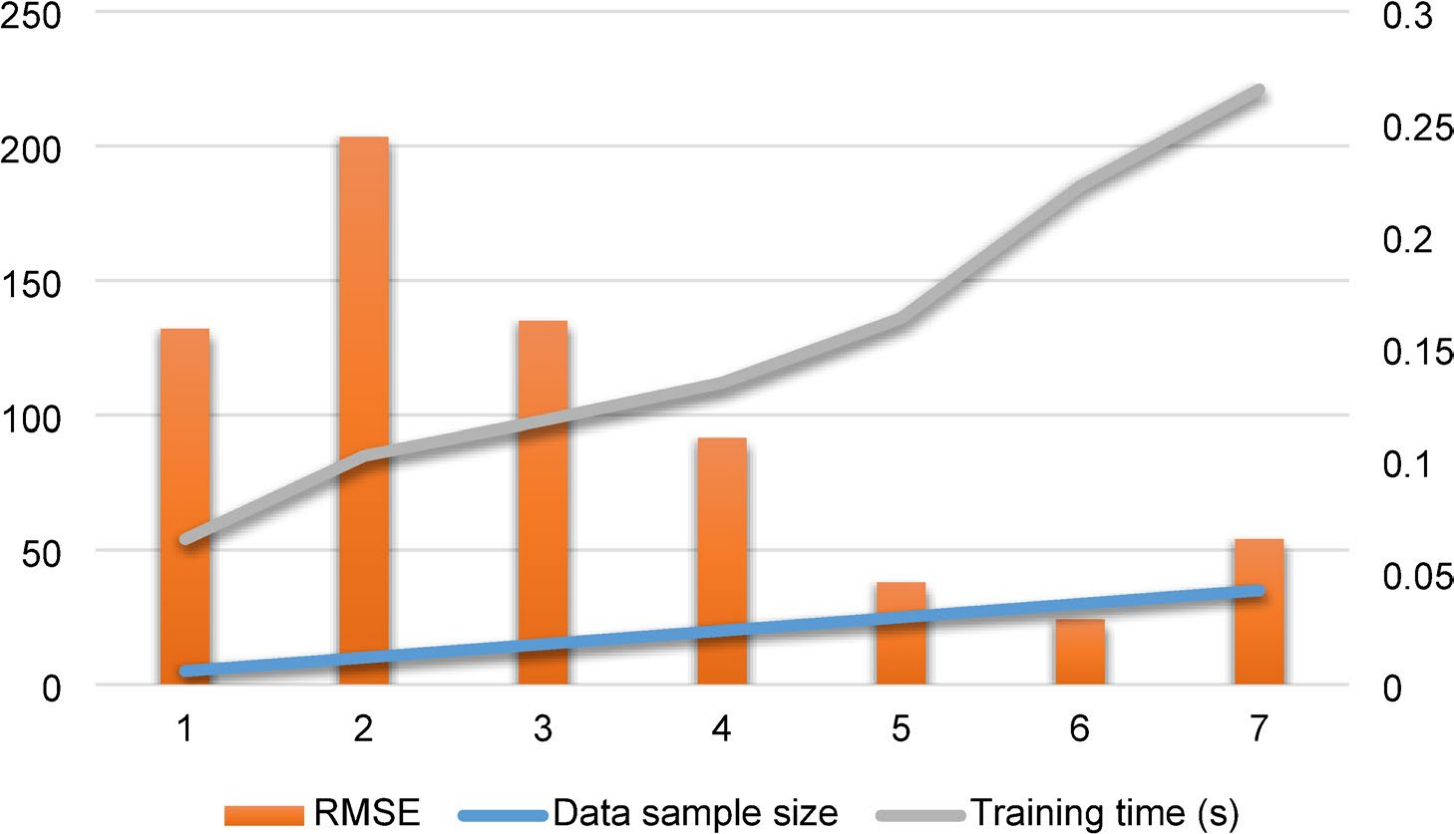
Trade-off between RMSE and training time considering data sample size.

Table 6 depicts the conducted analysis to select the 30 data samples from each charging cycle for different LIB parameters. A trade-off curve illustrating the relationship between RMSE and training time, based on various data features (5, 10, 15, 20, 25, 30 and 35), is depicted in Fig. 4. The trade-off curve involving RMSE and training time indicates that the careful choice of data features is essential. The computational complexity is low when the data features are minimal. It produces less accurate findings, though. On the contrary, adding additional data attributes increases computing complexity. As a result, choosing the right data characteristics is essential to achieving accurate results with the least amount of computational complexity.

**Table 6:** Determination of a suitable data sample size for the proposed model training.

Data sample size	Data dimension	RMSE	MSE	MAPE	Training time (s)
5	15	0.1584	2.347*10 − 4	0.1086	54
10	30	0.2440	4.498*10 − 4	0.1127	85
15	45	0.1619	2.63 × 10^− 4^	0.1125	98
20	60	0.1101	1.15*10 − 4	0.0385	112
25	75	0.0456	2.58 × 10^− 5^	0.0521	136
30	90	0.0292	8.55 × 10^− 6^	0.0152	185
35	105	0.0649	4.21*10–5	0.0248	221

**Fig. 5:**
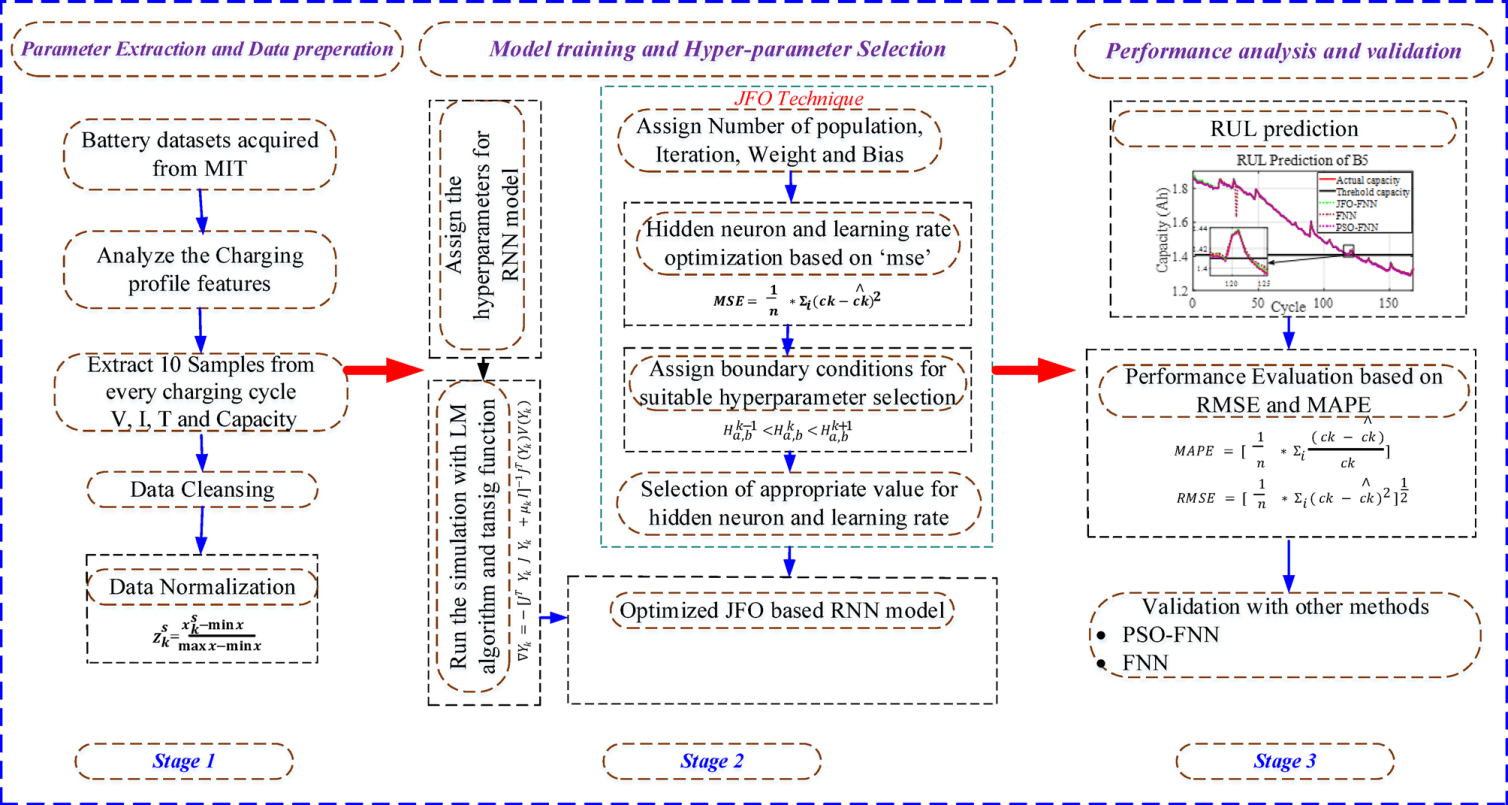
Proposed RUL prediction framework with hybrid JFO-FNN algorithm and MBMI profile.

The JFO-FNN method may be divided into three tiers, as shown in Fig. 5: data acquisition and preparation level, training level and lastly prediction level. The initial step is obtaining appropriate parameters by analyzing the LIB datasets. The LIB parameters such as VIT and discharge capacity are selected and further, the SS method is applied to construct a 91-dimensional data framework. The SS method suitably selects 30 data samples from VIT with a single data sample from capacity. The developed data framework is appropriately applied for carrying out the model training in the second level. During JFO-FNN testing for each LIB, the model training was performed on the dataset from the other three LIBs. The JFO-FNN model parameters such as iterations, epochs, and jellyfish population are attained using the trial-and-error method, however, the FNN model parameters, i.e. learning rate and hidden neurons are selected by utilizing the JFO technique. In the final stage, the RUL prediction is performed by analyzing the cycle number i.e. threshold capacity level. The JFO-FNN training is examined through error indices i.e. RMSE and MAPE which are equated as:

9 $$\:RMSE=\sqrt{\frac{1}{n}\sum\:_{n=1}^{n}{\left|ck-{ck}^{\varDelta\:}\right|}^{2}}$$

10 $$\:MAPE=\frac{1}{n}\sum\:_{n=1}^{n}\frac{\left|ck-{ck}^{\varDelta\:}\right|}{ck}$$

In addition, the RUL error is estimated with the true RUL and predicted RUL as shown as:

11 $$\:RULerror=RULpredicted-RULtrue$$

where, the negative $\mathop {RUL}\nolimits_{{error}}$denotes that $\mathop {RUL}\nolimits_{{predicted}}$is smaller than $\mathop {RUL}\nolimits_{{true}}$and vice versa.

## Results and discussion

A JFO-FNN-based RUL prediction framework is developed using different MBMI profiles. The constructed data framework is developed using different LIBs and their related parameter showing high correlation with the output capacity curve. Model training and testing are performed on different training and testing datasets. Table 7 shows the evaluation of the charging/discharging profile to select the best profile for LIB parameter selection. Due to the rapid current fluctuations and no preset protocols, the initial outcomes to predict the LIB show high error with the discharge profile as compared with the charging profile.

**Table 7:** Selection of the operational profile to select the lib parameters from nasa database.

Operational profile	Parameters used	RMSE	MAPE
Charging profile	Voltage, temperature and current	0.1930	0.0908
Discharging profile	Voltage, temperature and current	0.3540	0.1412

### Analysis of the LIB dataset from the NASA database

**Fig. 6:**
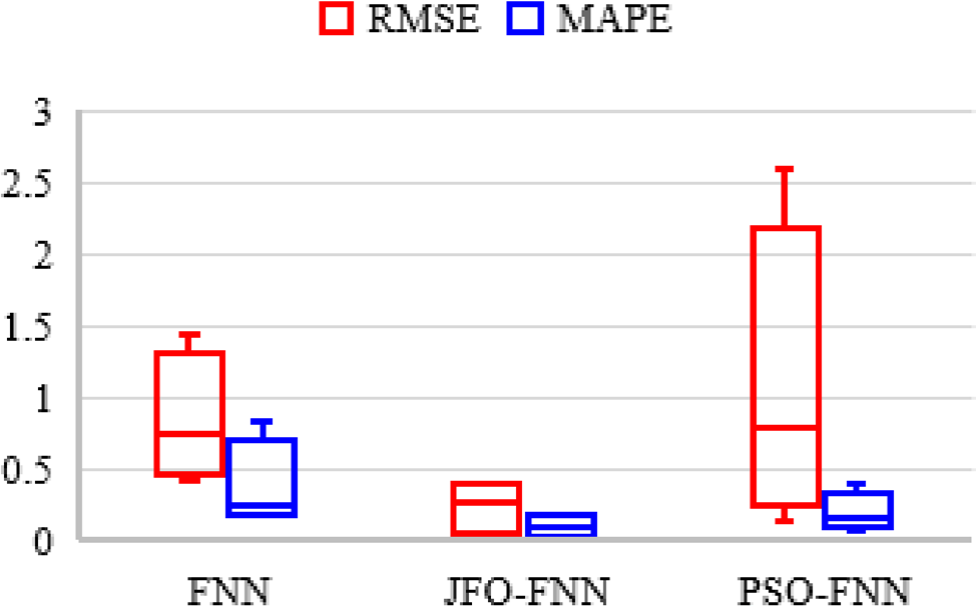
Box plot curve showing the error indices for the RUL prediction models with the NASA LIB datasets.

The JFO-FNN model is trained and validated with a traditional FNN model and PSO-FNN technique to predict the RUL of LIBs as presented in Table 8. In this work, each LIB dataset is regarded as a testing dataset, while the remaining three LIB datasets provide 70% of the training dataset. The RUL prediction is analyzed starting from cycle one. This is assumed as a complete single LIB dataset is applied for model testing, hence plots the capacity curve from cycle one. The initial capacity for B5, B6, B7 and B18 is assigned as 1.96, 2.04, 1.89 and 1.86 Ah whereas, the threshold capacity or the knee point for B5, B6, B7 and B18 is 1.41, 1.39, 1.51 and 1.41 Ah. The performance indicates that the JFO-FNN model consistently outperforms FNN and PSO-FNN in terms of accuracy and reliability for RUL prediction across all batteries. JFO-FNN achieves the lowest RMSE, MSE, and MAPE values, demonstrating superior predictive accuracy. For instance, in B5, JFO-FNN records an RMSE of 0.0292 compared to 0.5961 for FNN and 0.1299 for PSO-FNN. Additionally, JFO-FNN maintains minimal RUL errors and stable predictions. However, this improved performance comes with a trade-off in terms of computational cost. Specifically, the JFO-FNN model incurs a notably higher training time ranging from 173.84 to 192.74 s primarily due to the iterative nature and exploration–exploitation balance of the Jellyfish Optimization algorithm. Despite this increased training overhead, the inference latency of JFO-FNN remains competitive, with an average of ~ 0.46 milliseconds, which is only marginally higher than that of PSO-FNN and baseline FNN. This demonstrates that while JFO-FNN may not be ideal for real-time online learning scenarios, it is well-suited for offline training followed by real-time inference in battery management systems, where fast and accurate RUL predictions are essential. Figure 6 shows the box plot for different models executed with the NASA database. The box plot compares the performance of FNN, JFO-FNN, and PSO-FNN for RMSE and MAPE showing high variability, especially in PSO-FNN and the FNN model.

**Table 8:** Performance indices of libs with the Mcmi profile.

LIB	Executed algorithms	Performance indices					
		RMSE	MSE	MAPE	RUL error	Training Time (s)	Inference latency (ms)
B5	FNN	0.5961	0.0036	0.2912	0	0.2760	0.33
	JFO-FNN	0.0292	8.55 × 10^− 6^	0.0152	0	185.02	0.46
	PSO-FNN	0.1299	1.68 × 10^− 4^	0.0624	0	97.32	0.42
B6	FNN	1.4294	0.0204	0.8194	0	0.2636	0.32
	JFO-FNN	0.1171	1.37 × 10^− 4^	0.0358	0	173.84	0.45
	PSO-FNN	2.5783	0.0062	0.3918	0	77.32	0.41
B7	FNN	0.4100	0.0017	0.1671	0	0.2272	0.31
	JFO-FNN	0.3979	0.0016	0.1862	1	192.74	0.47
	PSO-FNN	0.5808	0.0034	0.1595	−2	56.70	0.40
B18	FNN	0.8929	0.0080	0.2043	2	0.2788	0.34
	JFO-FNN	0.3955	0.0016	0.1593	−1	175.31	0.46
	PSO-FNN	0.9887	0.0098	0.1643	0	72.53	0.41

**Fig. 7:**
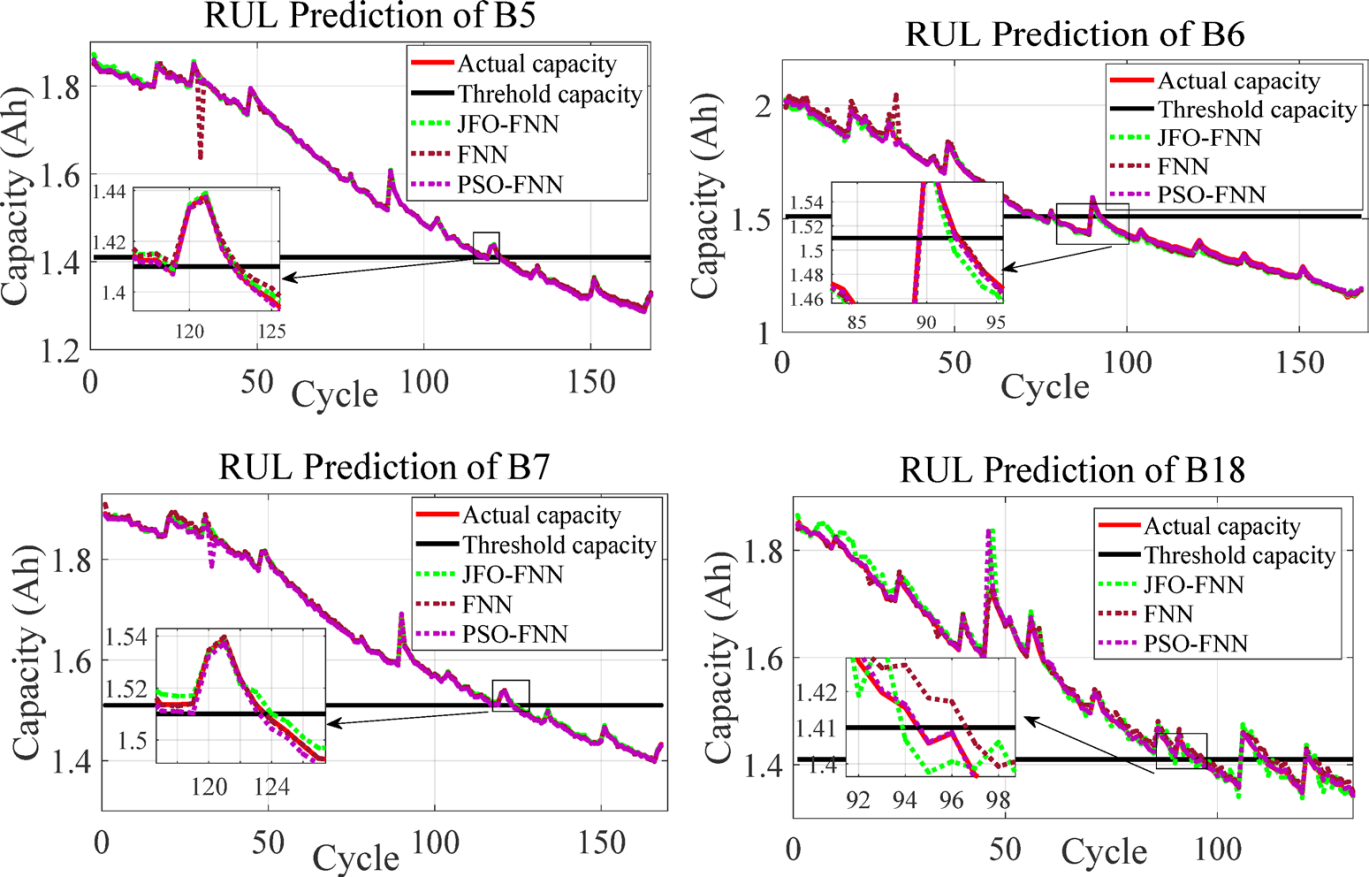
Degradation profile curve to predict the RUL for the LIBs with MCMI profile data.

Despite this, its improved accuracy justifies the increased computational cost, confirming JFO-FNN’s robustness in RUL prediction for the LIBs. The RUL prediction outcomes for LIB datasets are presented on Fig. 7. The degradation curve for every LIB demonstrates the effectiveness of the JFO-FNN model compared to standard FNN and PSO-FNN approaches.

In all cases, the JFO-FNN consistently provides predictions that closely align with the actual capacity trends, particularly during the critical phases near the threshold capacity. The inset highlights areas of rapid capacity degradation, showing that JFO-FNN outperforms other models by maintaining better accuracy and tracking. For B5, JFO-FNN captures the discharge capacity pattern with minimal deviation. Similarly, in B6 and B7, JFO-FNN maintains a tighter fit to the actual capacity, especially in regions with sudden drops. PSO-FNN and standard FNN show larger deviations, particularly in critical prediction phases. These results validate the JFO-FNN’s robustness and precision in forecasting RUL across diverse datasets, highlighting its ability to provide reliable predictions in both stable and fluctuating capacity cycles. Furthermore, the effect of capacity regeneration phenomena is more dominant in B6. Due to this, the error indices outcome for B6 was comparatively high compared with the other tested LIB datasets.

### Validation with the MIT-Stanford dataset

#### LIB datasets from batch ‘2017-05-12’

The JFO-FNN model is validated with the MIT-Stanford dataset from batch ‘2017-05-12’. The JFO-FNN model showed high accuracy and robustness during training with validated LIB datasets owing to the high volume of the training dataset. Table 9 shows the performance indices for the proposed JFO-FNN model, PSO-FNN model and conventional FNN model for different datasets obtained from the MIT-Stanford database. The cycle number for c33, c34, c35 and c36 is stated as 870, 842, 709 and 876 whereas, the initial capacity is mentioned as 1.0877, 1.0790, 1.0799 and 1.0946 Ah and the threshold capacity or the knee point is assigned as 0.94 Ah.

**Table 9:** Performance indices of libs with Mcmi profile for batch ‘2017-05-12’.

LIB	Executed algorithms	Performance indices					
		RMSE	MSE	MAPE	RUL error	Training Time (s)	Inference latency (ms)
C33	FNN	0.0658	4.33*10^− 5^	0.0369	0	0.35052	0.35
	JFO-FNN	0.0486	2.35*10^− 5^	0.0425	0	134.87	0.46
	PSO-FNN	0.1159	1.34*10^− 4^	0.0271	0	110.24	0.43
C34	FNN	0.0977	9.54*10^− 5^	0.0675	0	0.3639	0.36
	JFO-FNN	0.0201	4.04*10^− 6^	0.0153	−5	140.16	0.47
	PSO-FNN	0.0754	5.68*10^− 5^	0.0645	−45	150.17	0.45
C35	FNN	0.1566	2.45*10^− 4^	0.0730	−1	0.4248	0.37
	JFO-FNN	0.0815	6.64*10^− 5^	0.0720	−5	119.82	0.48
	PSO-FNN	0.2187	4.78*10^− 4^	0.1212	−3	154.67	0.44
C36	FNN	0.6472	0.0042	0.4530	−5	0.3688	0.34
	JFO-FNN	0.1839	3.38*10^− 4^	0.1256	1	130.93	0.46
	PSO-FNN	1.9902	0.0369	1.4658	−30	63.22	0.42

The performance indices for batteries C33–C36 from batch ‘2017-05-12’ indicate that the JFO-FNN model consistently surpasses both FNN and PSO-FNN in predictive accuracy and reliability. JFO-FNN achieves the lowest RMSE and MSE across all datasets, reflecting superior generalization and precision in RUL estimation. However, this comes at the expense of increased training time, with values ranging from approximately 119 to 140 s. This is significantly higher than the baseline FNN (sub-second) and PSO-FNN (ranging from 63 to 155 s), indicating that the JFO-based optimization introduces a considerable computational burden during model training. In contrast, inference latency for JFO-FNN remains within a narrow and acceptable range of 0.46–0.48 milliseconds only slightly higher than FNN and PSO-FNN demonstrating that once the model is trained, it can still deliver near-instantaneous predictions suitable for real-time deployment. This trade-off suggests that JFO-FNN is best leveraged in settings where offline training is feasible, and real-time inference accuracy is critical, such as in BMS applications for EVs.

**Fig. 8:**
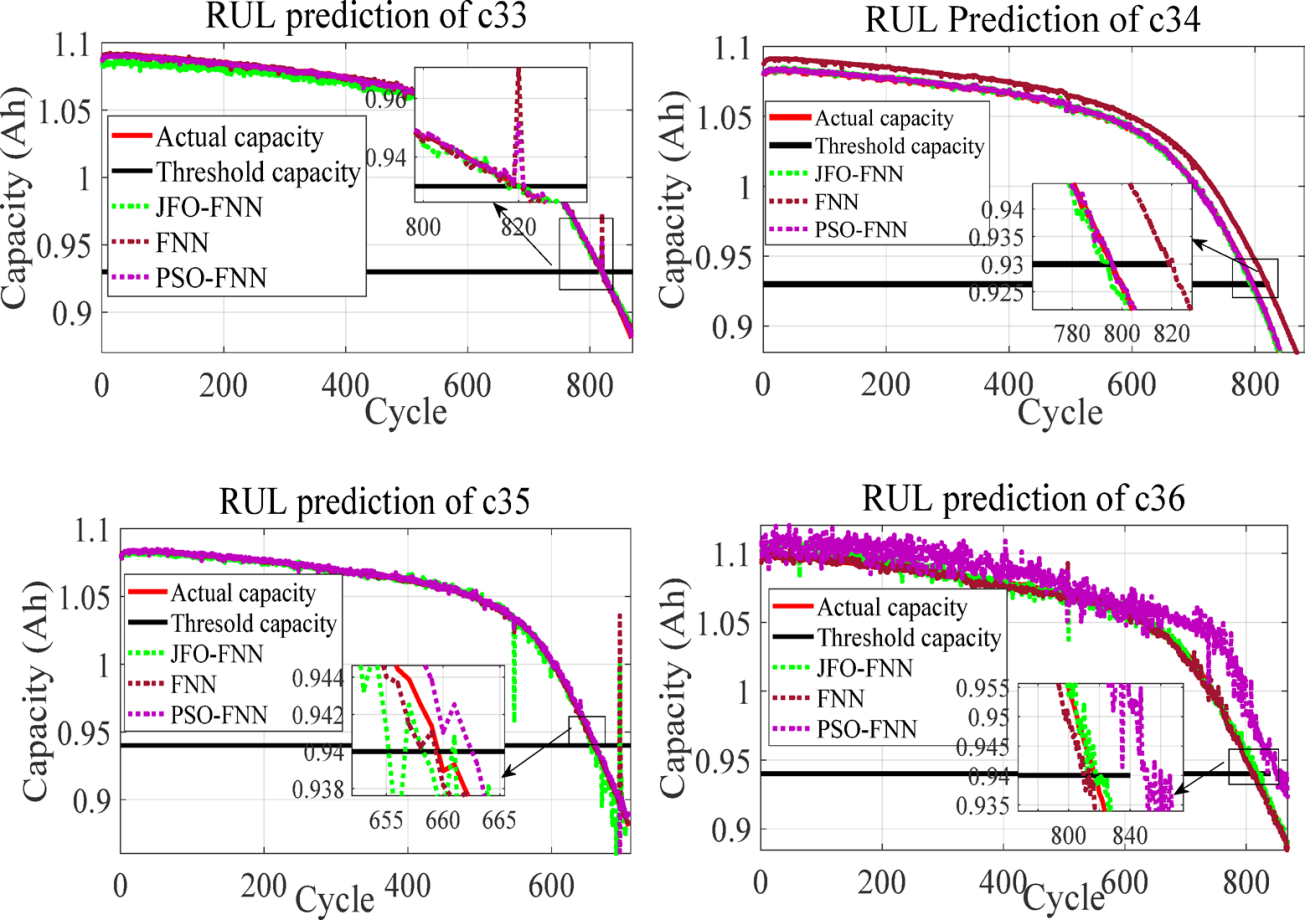
Degradation profile curve to predict the RUL for the LIBs from batch ‘2017-05-12’ with MCMI profile data.

Additionally, the prediction curve for the MIT-Stanford dataset is shown in Fig. 8. The JFO-FNN model was successful in reconstructing the capacity curve due to the right selection of the features and an optimized FNN model network. A high error was achieved with dataset C36 because of capacity regeneration phenomena, which resulted in inaccurate reconstitution of the predicted capacity curve as well. For instance, the RMSE value under C33, C34 and C35 with the JFO-FNN model was 0.0486, 0.0201 and 0.0815. However, the RMSE value with C36 was 0.1839 which is comparatively higher than other datasets because of capacity regeneration. The least execution time among the three models was taken by the traditional FNN model with an average time of 0.37s while the average execution time for the proposed JFO-FNN model is estimated as 131.445 s and the PSO-FNN model was 119.57 s respectively.

#### LIB dataset from batch ‘2018-04-12’

The JFO-FNN algorithm is trained and validated with datasets from other batch ‘2018-02-20’. The validation with other batches was conducted to demonstrate the applicability and effectiveness of the JFO-FNN model as shown in Table 10. The cycle number for the LIB cell c33, c34, c35 and c36 is stated as 2189, 824, 988 and 1027 whereas, the initial capacity is mentioned as 1.0650, 1.0452, 1.0601 and 1.0710 Ah and the threshold capacity or the knee point is assigned as 0.98 Ah for c33 and 0.93 Ah for c34, c35 and c36. The performance indices highlight the superior predictive accuracy of the JFO-FNN model compared to FNN and PSO-FNN for RUL estimation across all datasets. JFO-FNN consistently achieves lower RMSE, MSE, and MAPE values, indicating its robustness in minimizing prediction errors. Specifically, JFO-FNN outperforms in C34 and C35, where it demonstrates a significant reduction in error while maintaining stable RUL predictions. However, in case of c33, the JFO-FNN technique showed high error compared with other validated models demonstrating the need to use specialized feature techniques. The high error could be due to the capacity regeneration or irregular degradation pattern during experimental protocols. The training time for JFO-FNN ranges from approximately 150 to 224 s, which is notably longer than PSO-FNN (93–170 s) and much higher than the lightweight FNN (under 1 s in all cases). This highlights the added computational burden introduced by the JFO optimizer during model training. However, inference latency remains within a tight and practical range for all models, with JFO-FNN requiring 0.45–0.48 milliseconds, only marginally higher than FNN and PSO-FNN. Although FNN shows faster training times, its predictive accuracy is notably lower. PSO-FNN displays moderate performance but suffers from higher error rates, especially in complex datasets. These results underscore JFO-FNN’s efficiency in delivering precise RUL forecasts while effectively balancing computational complexity.

**Table 10:** Performance indices of libs with Mcmi profile for batch ‘2018-04-12’.

LIB	Executed algorithms	Performance indices					
		RMSE	MSE	MAPE	RUL error	Training Time (s)	Inference latency (ms)
C33	FNN	0.0720	5.18*10^− 5^	0.0545	−3	0.5038	0.35
	JFO-FNN	0.6973	0.0049	0.4801	−20	151.74	0.45
	PSO-FNN	0.9507	0.0090	0.8606	+ 60	118.31	0.42
C34	FNN	0.4069	0.0017	0.1409	−4	0.6628	0.36
	JFO-FNN	0.2877	8.27*10^− 4^	0.1066	0	223.55	0.47
	PSO-FNN	0.8363	0.0070	0.2211	−4	170.26	0.44
C35	FNN	0.8413	0.0071	0.4271	+ 1	0.8019	0.38
	JFO-FNN	0.2692	7.24*10^− 4^	0.1252	+ 1	150.33	0.48
	PSO-FNN	0.3791	0.0014	0.1711	+ 4	93.39	0.43
C36	FNN	0.1324	1.75*10^− 4^	0.1115	+ 6	0.4523	0.34
	JFO-FNN	0.0949	9*10^− 5^	0.0862	+ 12	190.57	0.46
	PSO-FNN	0.1385	1.91*10^− 4^	0.1074	+ 4	144.37	0.41

**Fig. 9:**
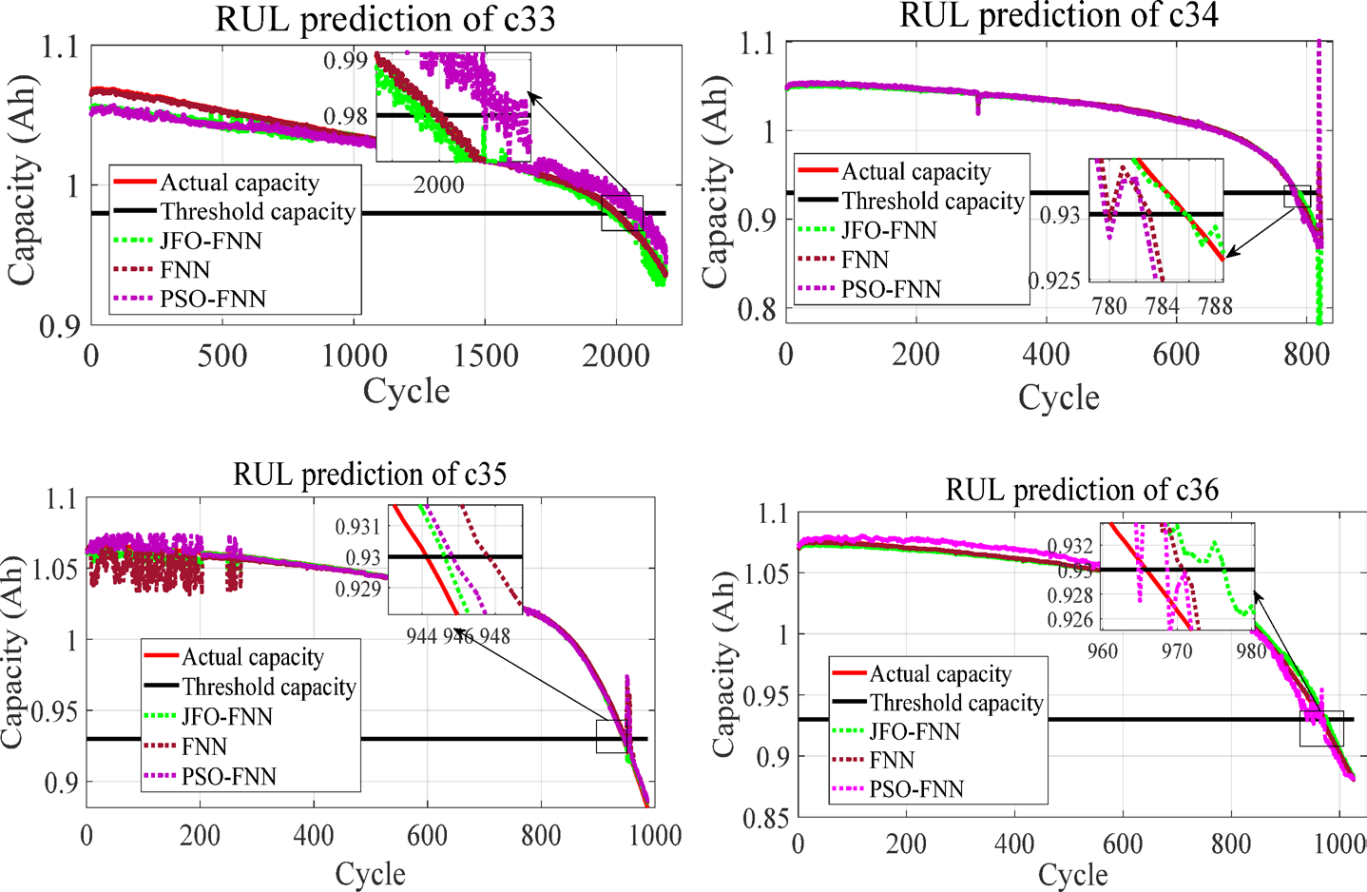
Degradation profile curve to predict the RUL for the LIBs from batch ‘2018-04-20’ with MCMI profile data.

The RUL prediction curves for batteries c33, c34, c35 and c36 from batch ‘2018-02-20’ illustrate the superior predictive accuracy of the JFO-FNN model over standard FNN and PSO-FNN as depicted in Fig. 9. JFO-FNN consistently aligns closely with the actual capacity, particularly near the critical threshold regions, as highlighted in the insets. For instance, in C33 and C34, JFO-FNN minimizes deviation during rapid degradation phases, outperforming other models in tracking dynamic capacity changes. In contrast, FNN and PSO-FNN exhibit significant errors and instability in high-degradation cycles, as evident in C35. These results confirm JFO-FNN’s robustness and precision in providing reliable RUL estimates across varying degradation profiles.

**Fig. 10:**
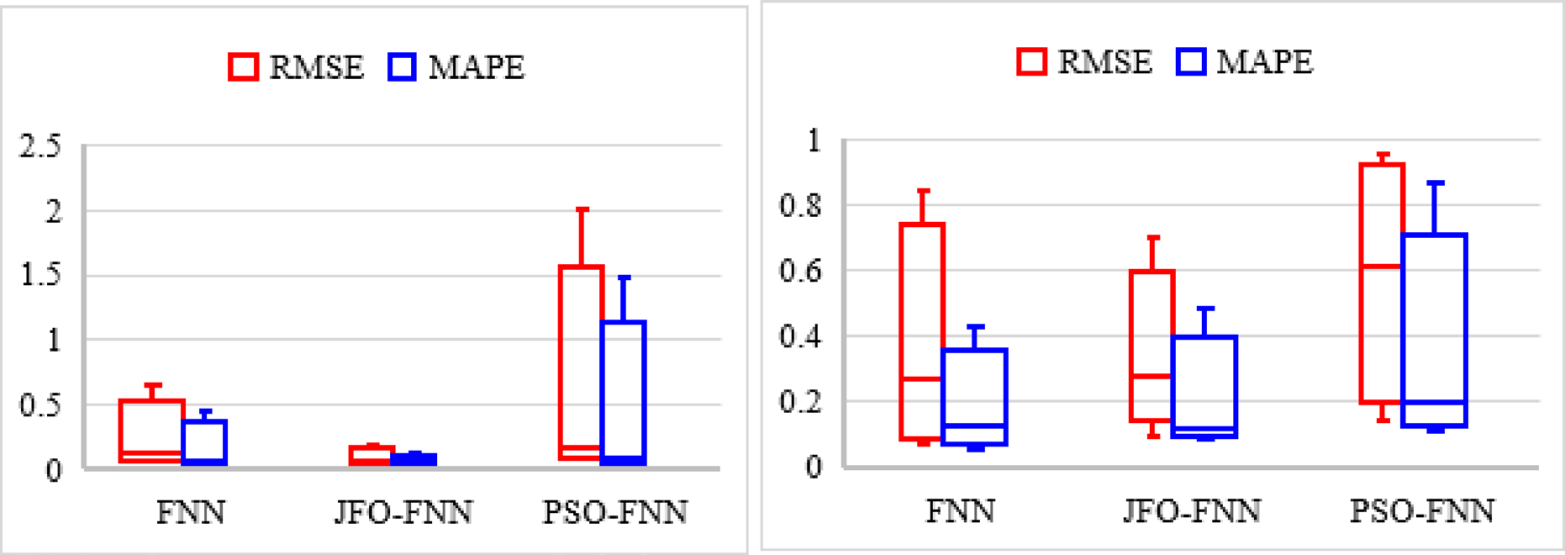
Box plot curve showing the error indices for the RUL prediction models with MIT-Stanford LIB datasets from batch ‘2017-05-12’ and batch ‘2018-04-12’.

Figure 10 illustrates the RMSE and MAPE distributions for FNN, JFO-FNN, and PSO-FNN using MIT-Stanford battery datasets from batches ‘2017-05-12’ and ‘2018-04-12’. The JFO-FNN exhibits the lowest errors, indicating better performance, while PSO-FNN shows higher variability, particularly in RMSE, suggesting instability in predictions. The average training time and inference latency of the trained JFO-FNN model were measured as 170 s and 0.46 ms per sample on an Intel i7 CPU, indicating suitability for real-time BMS deployment scenarios with sub-10 ms response requirements. From the above results, it is evident that selecting appropriate LIB parameters and identifying critical data features are essential for ensuring accurate predictions. In this study, a LIB parameter was selected from the charging profile and additionally, accurate selection of data samples was conducted to enhance prediction accuracy. It was observed that, with the NASA dataset, the error was primarily due to local fluctuations from capacity regeneration. Whereas the dynamic features of the LIB parameters with the MIT-Stanford dataset influenced prediction outcomes. By using the SS technique, it was possible to reconstitute the capacity curve. The rapid variation in parameter features, however, renders optimized models like JFO-BPNN incapable of reconstructing the estimated degradation curve.

Furthermore, the operational profile analysis in this work to extract the LIB dataset was limited to charging profile only and therefore, further research analysis could be performed to conduct a comprehensive analysis of the based on the discharging process. Moreover, other parameters such as time and impedance maybe acquired to train state of the art models for RUL prediction which remains unaddressed in this work.

### Comparative analysis of the outcome with other models

In this study, the JFO-FNN model is trained with the NASA dataset and tested using the MIT-Stanford dataset. Additionally, the results are compared with previously conducted research considering state-of-the-art models, as presented in Table 11. To provide an unbiased comparison, the LIB dataset B5 was considered. The analysis is performed by considering execution factors such as model implementation, error indices (i.e., RMSE), and existing research gaps. A deep neural network (DNN) model was applied which delivered satisfactory outcomes with the RMSE of 3.427^29^. In another work^30^, optimized SVR model was applied for RUL prediction which shows the RMSE as 0.0307. Ansari et al.^15^ utilized the BPNN model which attains a higher RMSE as 0.0819 due to the inappropriate selection of model parameters. A stacked autoencoder (SAE) model was used for RUL prediction which delivered the RMSE as 0.65 depicting high error. With the GRU model^31^, the RMSE achieved was 0.97% which could be further improved with the utilization of the right amount of data and suitable model parameters.

**Table 11:** Comparitive analysis of proposed Rul prediction framework with models.

Reference	Algorithms	Model complexity	RMSE	Adaptability to noise and unseen conditions	Research gap
^29^	DNN	3 hidden layers × 128 neurons; Total Parameters: ~34,945	3.427	Moderate	-The model parameter selection was not conducted comprehensively
^30^	Ant lion-SVR	Trainable parameter: 1000–2000	0.0307	Low	-The LIB parameters such as voltage, current, impedance was not critically analyzed and used
^15^	BPNN	1 hidden layer with 10 neurons, learning rate as 0.005, iteration as 20 and epoch as 1000	0.0819	Moderate	-The BPNN model parameters selection was not accurate and thus the error was high
^32^	SAE	3 hidden layers	0.65	High	-The model demonstrated computational complexity with high error
^33^	LSTM	Batch size 32, epoch 500, 3 hidden layers, hidden neuron as 128, trainable parameters as 331,393	5.6262	High	-Use of suitable techniques to extract the data samples could be performed
^31^	GRU	Hidden layer as 64 neurons, dropout as 0.2, trainable parameters as 12,737	0.97%	Moderate	-High-dimensional data could be applied for training the model
**Proposed**	**JFO-FNN**	**Iteration 100**,** epochs as 1000**,** trainable parameters vary between [7 241]**	**0.0292**	**High**	**-Discharge profile of the LIB can be analyzed in future research works**

## Conclusion

This work proposes an integrated RUL prediction framework comprising the FNN model and JFO. The JFO-FNN model is trained with key LIB parameters, which strongly shows a correlation with the output. are extracted. A data framework with high dimensionality, consisting of 91 data features, is constructed. The training of the JFO-FNN model with developed data with the MBMI profile outperforms the PSO-FNN model and conventional FNN models considering different error indices. With B5, the MSE, RMSE, and MAPE are 3.9494 × 10⁻⁴, 0.1987, and 0.0828, respectively, which are significantly more accurate compared to 2.4646, 0.0607, and 1.1752 in B6. Compared with the PSO-FNN model and conventional FF model, the proposed JFO-FNN model is more accurate and has better capacity reconstitution ability. Further, the capacity regeneration effect showed high influence in the LIB such as B6 and B18, respectively, delivering a higher error in terms of MSE, MAPE and RMSE compared to the other LIBs such as B5 and B7. The JFO-FNN model is validated with the MIT-Stanford dataset and shows good results based on low error and high accuracy. The suggested work would facilitate regulated management by substituting the LIBs before reaching the threshold of RUL prediction.

It is important to note that this study primarily focuses on the implementation and evaluation of the JFO algorithm for hyperparameter tuning in battery RUL prediction models. While the findings highlight the algorithm’s effectiveness in improving predictive accuracy, training time, and inference latency, the scope of this work does not extend to real-time deployment scenarios in production EV battery management systems. Future research will address scalability and integration challenges in embedded environments to evaluate the algorithm’s performance under real-time constraints. This work could be further extended to LIB applications for charging/discharging in future EVs and LIB swapping stations. Furthermore, the integration of JFO with advanced hybrid architectures (e.g., recurrent–convolutional models) and domain-specific feature engineering will be explored to enhance RUL prediction performance further. Lastly, the parameter selection should be carefully analyzed to achieve more accurate prediction outcomes.

## Data Availability

The datasets used and/or analyzed during the current study are available from the corresponding author upon reasonable request.
